# Comparative Proteomic Analysis of *Acremonium chrysogenum* Strains: Key Changes Converting the Wild-Type Strain into Antibiotic Cephalosporin C Biofactory

**DOI:** 10.3390/jof11110822

**Published:** 2025-11-20

**Authors:** Alexander A. Zhgun, Maria V. Dumina, Alexey V. Beletsky, Arthur T. Kopylov, Viktor G. Zgoda

**Affiliations:** 1 Federal Research Center “Fundamentals of Biotechnology” of the Russian Academy of Sciences, Leninsky Prosp. 33-2, 119071 Moscow, Russia; duminamaria@gmail.com (M.V.D.); mortu@yandex.ru (A.V.B.); 2 Institute of Biomedical Chemistry RAMS, Pogodinskaya St. 10, 119121 Moscow, Russia; a.t.kopylov@internet.ru (A.T.K.); victor.zgoda@gmail.com (V.G.Z.)

**Keywords:** *Acremonium chrysogenum*, cephalosporin C, antibiotic production, fermentation, secondary metabolites of fungi, high-yielding strain, comparative proteomic analysis, differentially expressed proteins (DEPs), biosynthetic gene clusters (BGCs)

## Abstract

*Acremonium chrysogenum* is the only industrial producer of the antibiotic cephalosporin C (CPC), the starting substance for manufacturing cephalosporins of the first to fifth generations. Strains produced for industrial use are significantly improved by multiple rounds of random mutagenesis; however, the molecular basis for such changes is not fully understood. In this study, we attempt to elucidate key changes that occurred at the proteome level in the CSI program of *A. chrysogenum* HY (RNCM F-4081D), with CPC production 300-fold higher than that in the parental *A. chrysogenum* WT strain (ATCC 11550). Our work reveals that more than 30% of proteins are differentially expressed at different stages of fermentation. Among the identified changes, the most critical appears to be upregulation of beta-lactam biosynthetic enzymes. The data also suggest shifts in the primary metabolic pathways, providing building blocks for beta-lactam synthesis reactions, including the amino acid precursors cysteine and valine and the substrate for the expandase reaction, α-ketoglutarate. Changes in energy flows in favor of targeted metabolic pathways are also revealed. High-yielding CPC production appears to be accompanied by oxidative stress, as key oxidative stress enzymes are upregulated. Our findings are consistent with previous investigations describing changes that occurred in other fungal strains improved by classical methods. This points to general key changes leading to high-yield production. A deeper understanding of these features is important for predicting the target effects of improved industrial producers of secondary metabolites.

## 1. Introduction

The high-yielding *Acremonium chrysogenum* HY strain (RNCM F-4081D), modified via the classical strain improvement (CSI) program, is one of the most comprehensively characterized cephalosporin C (CPC) producers in terms of both phenotypic and genetic changes [1,2]. This strain was obtained after multiple rounds of mutagenesis and selection from the wild-type strain *A. chrysogenum* WT (ATCC 11550), isolated by Giuseppe Brotzu from seawater off the coast of Sardinia, Italy [3,4]. *A. chrysogenum* WT, like other wild-type fungal strains, produced insufficient amounts of the target antibiotic for industrial use [5,6]. The production level of *A. chrysogenum* WT is several tens of mg of CPC per liter; its high-yielding descendant *A. chrysogenum* HY has an increased cephalosporin C yield, exceeding 10 g per liter during laboratory fermentation in shake flasks [7]. The CSI program resulted in 3730 mutational events, 56 of which led to major disruptions in the encoded proteins [2]. One of the most important results of the CSI program for *A. chrysogenum* HY was an increase in the expression of beta-lactam biosynthesis genes by 10–300 times or more [7]. Such upregulation occurred without accompanying an increase in gene dosage (duplications) and translocations of the “early” and “late” biosynthetic gene clusters (BGCs) of beta-lactams to other chromosomes [7,8].

The increase in CPC production was accompanied by a number of phenotypic changes, some of which were directly related to the biosynthesis of this antibiotic or the reprogramming of cell resources in favor of the targeted biosynthetic pathway; others may have been the result of side mutations that occurred in the CSI program. In *A. chrysogenum* HY decreased vitality was expressed as a decrease in the size of colonies during cultivation on an agar nutrient medium [9], a decrease in biomass during submerged fermentation [7], a reduction in conidiation [10] and thinning of the cell wall [11]. The reduction in colony size and biomass may be due to the toxicity of high-yield production when the resources needed for growth are partially redirected for targeted secondary metabolism. The reduction in conidia formation may also be associated with high-yield production, as in *A. chrysogenum* HY, at the final stages of fermentation, the mycelium fragments into dumbbell-shaped arthrospores (or oidia) that actively produce CPC [12], whereas in WT, the primary morphological forms are resting conidia, which do not produce CPC. On the other hand, a reduction in conidia formation significantly reduces the viability of the strain. This strain cannot be stored for a long time at low temperatures; standard freezing procedures with cryoprotectants lead to cell death. The only effective way to store it is cultivation on agarized media, with reseeding every 2–3 months. The inability to obtain viable *A. chrysogenum* HY cells after low-temperature freezing is primarily due to the loss of the ability to form conidia, which tolerate long-term storage much more effectively than mycelium [13,14,15,16]. Thinning of the cell wall may promote high-yield production by enhancing the transport of CPC out of the cell. However, this makes it difficult to obtain protoplasts for HY strain transformation. To introduce genetic material for experiments, a system based on *Agrobacterium tumefaciens*-mediated transformation (ATMT) was developed [10].

*A. chrysogenum* HY also lost the yellow-cream color, characteristic for *A. chrysogenum* strains and associated with the biosynthesis of sorbicillinoids, the polyketide secondary metabolites [17]. Similar decolorization of improved fungal strains has also been described for *Penicillium chrysogenum* Wisconsin 54-1255 and its derivatives, which are producers of penicillin G (PenG) [18,19]. This may be due to the fact that pigments, unlike many other secondary metabolites, are synthesized not in response to external signals but systematically, during the transition from trophophase to idiophase, which consumes the cell’s resources [20,21]. Inactivation of pigment biosynthesis allows the release of additional cellular resources for targeted metabolism [6]. In *P. chrysogenum* that bleaching was caused by a single Leu146Phe missense mutation in the polyketide synthase SorA (Pc21g05080), resulting in transcriptional silencing of the entire sorbicillinoid biosynthetic gene cluster (BGC) [19]. In this regard, the detected missense mutation Ile2397Lys in the gene of another polyketide synthase SorB is considered to be the cause of discoloration of the *A. chrysogenum* HY [2].

In *A. chrysogenum* HY, cellular energy flows are also redistributed in favor of the target metabolism. For this strain reduced activity of cytoplasmic membrane H^+^-ATPase (PMA) [22] facilitated increased CPC production [23]. PMA is the main enzyme consuming ATP in fungi, accounting for 20–50% of consumption [24,25]. The biosynthesis of cephalosporin C is associated with the consumption of ATP; for example, in the first stage, during the work of NRPC, three ATP molecules are consumed for the synthesis of LLD-ACV tripeptide—δ-(L-α-aminoadipoyl)-L-cysteinyl-D-valine [23]. In *A. chrysogenum* HY, cellular ATP content is significantly reduced compared to the wild-type parent strain [22,23]. A sequential increase in PMA activity in recombinant *A. chrysogenum* HY/PMA1 clones resulted in a correlated decrease in ATP content, CPC, and downregulation of BGC beta-lactam genes [23]. Another series of experiments showed that the MFS transporter CefT from the “early” cluster of beta-lactams is responsible for the transport of intermediates of cephalosporin C biosynthesis, such as penams (penicillin N, isopenicillin N) and cephems (deacetoxycephalosporin C, deacetylcephalosporin C, DAC) [26].

Another set of experiments revealed changes in polyamine metabolism in *A. chrysogenum* HY. This change is likely a side effect of random mutagenesis (discussed below) and affects CPC biosynthesis. Intracellular levels of major polyamines such as 1,3-diaminopropane (1,3-DAP) and spermidine (SPD) increased 4–5-fold compared to the wild-type strain [27]. This may be one of the reasons for the unexpected phenomenon in which the attenuated *A. chrysogenum* HY strain exhibits increased resistance to inhibitors of the key enzyme of polyamine biosynthesis, ornithine decarboxylase (ODC), compared to the more viable *A. chrysogenum* WT strain [27]. The addition of ODC inhibitors such as α-difluoromethylornithine (DFMO) or 1-aminooxy-3-aminopropane (APA) at 5 mM resulted in complete growth inhibition of WT, but not the HY strain. When cultured on agarized complex medium, the addition of polyamines led to earlier development of the characteristic pigment coloration in *A. chrysogenum* WT, caused by the biosynthesis of sorbicillinoids, against the background of its expected absence in *A. chrysogenum* HY [9]. Stimulation of sorbicillinoid biosynthesis in *A. chrysogenum* WT showed that exogenous administration of polyamines can intensify secondary metabolite pathways [28]. A similar effect, an increase in the intensity of pigment coloration with the addition of 1,3-DAP or SPD, was observed on a solid medium during the cultivation of Wisconsin 54-1255 [29]. The authors stated that the yellow-green coloration was caused by the secondary metabolite chrysogenin [29,30].

During submerged fermentation of *A. chrysogenum* HY on a complex medium, the addition of 1,3-DAP and SPD resulted in a 10–15% increase in CPC production and the upregulation of beta-lactam biosynthesis genes [9]. However, on a specially selected medium during submerged fermentation, adding polyamines was observed to have the opposite effect. The addition of 1,3-DAP resulted in a 12–15% increase in CPC, while addition of SPD resulted in a 15% decrease in CPC production [31]. Such differential effects are associated with the intersection of the pathways of CPC biosynthesis and polyamine catabolism, which uses one common substrate, acetyl coenzyme A (acetyl-CoA). Since N^1^-acetylation in the polyamine catabolic pathway is significantly more efficient for SPD than for 1,3-DAP, adding SPD, but not 1,3-DAP, depletes cytoplasmic acetyl-CoA, reducing the efficiency of acetylation in the final step of the target biosynthetic pathway, decreasing CPC and accumulating its precursor DAC [32]. Therefore, the total content of cephems, when adding both 1,3-DAP and SPD to a specially selected medium, increases approximately equally, by 10–15%. A similar effect of exogenous polyamines, leading to an increase in the yield of target secondary metabolites in fungal strains improved by classical methods, was also observed in association with the production of PenG in *P. chrysogenum* [29,33] and lovastatin in *Aspergillus terreus* [34]. The molecular basis for the effects induced by polyamines is still unknown. One hypothesis suggests that polyamines may stimulate pantothenic acid biosynthesis, the products of which activate core megasynthases of secondary metabolism such as nonribosomal peptide synthetases (NRPSs) or polyketide synthases (PKSs) [28]. On the other hand, the addition of polyamines upregulates the *laeA* gene for the global regulator of fungal secondary metabolism, which is a positive regulator for BGCs of beta-lactams and lovastatin [29,34,35]. The observed increase in polyamine content for *A. chrysogenum* HY may have been due to the CSI technique based on random mutagenesis, since it allowed clones to survive after sublethal exposures due to DNA protection from emerging free radicals [36,37] and/or participation in homology-directed DNA repair processes [38]. An increase in polyamine content leads to additional consumption of cellular resources. Since polyamine homeostasis in the cell, which is regulated by a feedback mechanism [39], is tightly controlled, we hypothesize that the addition of polyamines reduces their endogenous synthesis, leading to the release of additional resources for the synthesis of target metabolites and an increase in their production [27].

Previously, a comparative proteomic analysis was performed for a number of high- yielding fungal producers of secondary metabolites using two-dimensional gel electrophoresis. In particular, this method was used to characterize changes in the proteomes of (i) the high-yielding penicillin G producer, *Penicillium chrysogenum* AS-P-78, compared to the initial wild-type strain *P. chrysogenum* NRRL 1951 and *P. chrysogenum* Wis 54-1255, an intermediate strain in the CSI program [40] and (ii) the high-yielding CPC producer *A. chrysogenum* 84-3-81-41, compared to the initial wild-type strain *A. chrysogenum* ATCC 11550 [41]. For the *P. chrysogenum* Wis 54-1255 strain, comparative two-dimensional gel electrophoresis was also performed after fermentation with the addition of 1,3-DAP and SPD and without additives (control) [42]. The obtained comparative data made it possible to identify a number of changes at the level of enzymes of primary and secondary metabolism, cellular energy flows, stress response, etc., which enriched our understanding of the processes required for high-yielding production [43,44,45,46].

To explain and interpret the numerous available data on the genetics, biochemistry, and physiology of the high-yielding producer of cephalosporin C, strain *A. chrysogenum* HY, we conducted a comparative proteomic analysis relative to the parental strain of the wild-type *A. chrysogenum* WT. We also conducted a comparative analysis to identify changes occurring in individual strains (*A. chrysogenum* WT or HY) during fermentation. For this, we used comparative data analysis methods such as cluster analysis, MA plots, Venn diagrams, principal component analysis, and GO enrichment analysis. This allowed us to focus our attention in the next step on key changes at the proteome level that occurred in the CSI program and convert the wild-type strain into a CPC biofactory. Our studies have shown that *A. chrysogenum* HY undergoes a significant reprogramming of cellular resources for high-yield CPC production. The central change involves a significant upregulation of CPC biosynthetic enzymes by up to 50-fold or more. The content of some enzymes reaches 1–3% of the cellular proteome. These enzymes (PcbC and CefEF) require oxygen for their function, which explains, among other things, the need for good aeration during *A. chrysogenum* fermentation for high-yield CPC production. This may also explain the significant upregulation of oxidative stress proteins of the main categories (superoxide dismutase, proteins of the thioredoxin system, proteins of the glutathione system, and catalases) found in *A. chrysogenum* HY. The strongest upregulation, with protein content accounting for up to 13% of the proteome composition, was observed for peroxiredoxin, a component of the thioredoxin oxidase system. Comparative proteomic analysis also showed a reprogramming of primary metabolism flows in favor of the construction of precursor compounds for CPC biosynthesis, the amino acids L-cysteine and L-valine, which are required for the construction of the core of beta-lactams, and α-ketoglutarate, which is necessary for the conversion of penams to cephems. There was also an intensification of energy flows for CPC production due to changes in the ratios between enzymes of the pentose phosphate pathway, glycolysis, and the tricarboxylic acid cycle. In the final stages of fermentation, *A. chrysogenum* HY, unlike WT, did not exhibit conidiation-specific protein, characteristic of conidia formation, and did not accumulate excess quantities of a protein called the glucose-repressible gene, the appearance of which is associated with the aging of the fungal cell. After 120 h of fermentation, this protein constituted up to 37% of the WT strain proteome and was not detected in *A. chrysogenum* HY. A number of key changes at the proteome level in *A. chrysogenum* HY discovered in our work correlate with alterations that occurred in other beta-lactam producers in the CSI programs. Understanding such events is important for converting the wild-type strain into a biofactory for the production of secondary metabolites needed for industry and the targeted creation of high-yield producers using genetic engineering methods.

## 2. Materials and Methods

### 2.1. Materials

Triethylammonia hydrocarbonate was from Sigma, St. Louis, MO, USA; TCEP (tris-(2-carboxiethyl)-phosphine) was from Thermo Scientific, Rockford, IL, USA; DTT (dithiothreitol) was from Thermo Scientific, Canada; 4-vinylpyridine was from Sigma, St. Louis, MO, USA; 2-propanol was from Fluka, St. Louis, MO, USA; grade modified trypsin was from Promega, Madison, WI, USA; formic acid was from Merck, Darmstadt, Germany; methanol was from Fisher Chemical, Loughborough, UK.

### 2.2. Strains of Microorganisms

The following strains of *A. chrysogenum* were used in the work: (i) ATCC 11550 (*A. chrysogenum* WT, wild type isolate Brotzu, [4]) and (ii) RNCM F-4081D (*A. chrysogenum* HY, a high-yielding producer of the antibiotic cephalosporin C, obtained on the basis of *A. chrysogenum* WT [1]).

### 2.3. Fermentation of A. chrysogenum Strains

For submerged fermentation, *A. chrysogenum* strains routinely cultured on agarized complex (CPA) medium (40 g/L maltose, 10 g/L peptone, 20 g/L malt extract, 25 g/L agar, pH 7.0–7.4) were transferred to agarized slants with LPE medium (10 g/L glucose, 20 g/L yeast extract, 15 g/L NaCl, 10 g/L CaCl_2_, 25 g/L agar, pH 6.8) and incubated at 28 °C for 10–20 days. Fungal cells were collected with 10 mL 0.9% NaCl, transferred to 65 mL of the defined (DP) medium (28 g/L yeast extract, 28 g/L malt extract, 10 g/L peptone, 4 g/L chalk, 20 g/L soybean oil, pH 7.2) in 750 mL Erlenmeyer flasks, and incubated on a rotary shaker at 230 rpm at 28 °C. After 48–72 h of growth, 10 mL of culture was inoculated in 65 mL of complex (CP) medium (105 g/L corn extract, 60 g/L corn dextrin, 20 g/L corn starch, 3 g/L KH_2_PO_4_, 5 g/L glucose, 3.5 g/L MgSO_4_, 14 g/L (NH_4_)_2_SO_4_, 11 g/L chalk, 20 g/L soybean oil; supplemented with microelements: 18 mg/L CuSO_4_ × 5H_2_O, 150 mg/L ZnSO_4_ × 7H_2_O, 30 mg/L MnSO_4_ × 7H_2_O, 70 mg/L FeSO_4_ × 7H_2_O, pH 6.2–6.4). Fermentation was performed in 750 mL Erlenmeyer flasks for 120 h (230 rpm) at 28 °C for the first 24 h and at 24 °C for the rest of the process. Samples were collected after 72, 96, and 120 h of fermentation.

### 2.4. Extraction of Proteins from A. chrysogenum Cells

Proteins were extracted as described previously with some modifications [40]. Fungal cells were collected after cultivation on liquid complex (CP) medium, with or without the addition of polyamines, washed three times with 20 volumes of 10 mM potassium phosphate buffer (pH = 7.4), frozen in liquid nitrogen, and ground into a fine powder in a pre-cooled mortar in the presence of liquid nitrogen. The prepared powder from fungal cells was added with 10 mM potassium phosphate buffer (pH = 7.4) containing 0.1% (*w*/*v*) DTT and a cocktail of protease inhibitors (Merck, Lebanon, New Jersey, USA), incubated for 2 h at 4 °C, and the extract was clarified twice by centrifugation at 10,000 g for 10 min. Proteins were precipitated by adding 1/10 volume of 100% trichloroacetic acid (TCA) for 1 h at −20 °C.

### 2.5. Sample Preparation

The pellets after TCA precipitation were washed twice with cold ethanol to remove traces of TCA, dried under vacuum at 45 °C for 60 min and reconstituted in 75 mM triethylammonium bicarbonate (Sigma, St. Louis, MO, USA), 6% acetonitrile (HPLC grade, filtered for 0.2 µm, (Fisher Chemical, Loughborough, UK) and 0.25% sodium deoxycholic acid (Sigma, St. Louis, MO, USA). Protein solutions were heated at 95 °C for 5 min (with Thermomixer Comfort, Eppendorf, Germany) to enhance denaturation. After the cooling/freezing procedure, 3 mM TCEP (tris-(2-carboxyethyl)-phosphine) (Pierce™ Thermo Fisher, Rockford, IL, USA) was added to reduce the sulfhydryl bonds of cysteine residues and then incubated at 40 °C for 30 min with continuous vigorous stirring. After this, 2% solution of 4-vinylpyridine (Sigma, St. Louis, MO, USA) in 30% 2-propanol (Fisher Chemical, Loughborough, UK) was added to 0.2% finally for proteins alkylation. Mixture was incubated at 35 °C for 45 min in darkness. Proteins were digested with sequencing grade modified trypsin (Promega, Madison, WI, USA). For this purpose trypsin (400 ng/µL) was added at 1:50 ratio (*w*/*w*) sequentially in two steps. The enzymatic reaction was carried out at 37 °C for 9 h with repeated stirring for 3 min every 15 min to precipitate condensate from the walls of the test tube and terminated by adding formic acid to a final concentration of 1%. The resulting solutions were centrifuged at 12,000 rpm for 10 min at 20 °C to precipitate insoluble deoxycholic acid. Supernatant was diluted up to 250 µL by distilled water (Milli-Q Intergral-3 system, 18.5 mΩ*cm_2_, TOC < 3 ppb) and transferred into 3MWCO polyethersulfon spin filter tubes (Sartorius Stedium, Goettingem, Germany) and centrifuged for 45 min at 12,000 rpm at 25 °C. The resulting filtrate was diluted by 1% formic acid up to 500 µL and loaded onto the Discovery DSC-18 cartridges (Supelco, Supelco Park, Bellefonte, PA, USA), preconditioned with methanol and 0.5% formic acid for solid-phase extraction. Peptides were eluted by 1 mL of methanol with 3% formic acid (Acros Organics, Geel, Belgium) and dried under vacuum at 30 °C for 30–40 min (Eppendorf Concentrator Plus, Eppendorf, Germany). The resulting dried pellet was resuspended in 20 µL of 0.1% formic acid and transferred into glass inserts with polymer feet (Agilent, Santa Clara, CA, USA) for further LC-MS analysis.

### 2.6. Liquid Chromatography and Mass Spectrometry

The peptide samples were analyzed using a high-resolution Q Exactive mass spectrometer (Thermo Scientific, Waltham, MA, USA). The instrument was operated in positive ionization mode and equipped with Nanospray Flex NG ion source (Thermo Scientific, Waltham, MA USA). Mass spectra were acquired with a resolution of R = 70K (normalized to *m*/*z* 400) for precursor ions in a scan range of *m*/*z* 400–1200 and R = 17.5K (at *m*/*z* 400) for fragment ions. Following the MS survey scan, top-20 most abundant ions were triggered for fragmentatin and tandem scanning. Peptide fragmentation was performed using high-energy collision dissociation (HCD) mode with an activation energy of 27 arbitrary units (stepped ±25%) and collision gas (nitrogen) was 8 mTorr. Precursor ions were isolated within ±1 Th window and the first mass of fragmentation spectra was set to 210 *m*/*z*.

Precursors were dynamically excluded from targeting for 15 s after three sequential scans. Ions with charge states z = 1+, z > 5+ and ions with undefined charge states were excluded from triggering MS/MS scans. Peptides separation was performed on an Ultimate 3000 nano-flow HPLC system (Thermo Scientific, Waltham, MA, USA). Prior to chromatography analysis, peptides were trapped onto enrichment PepMap C18 column (0.5 mm inner diameter, 3 mm length, 5 µm particle size) using solvent C (2% acetonitrile, 0.08% formic acid, 0.015% trifluoroacetic acid). Chromatographic separation was carried out on an analytical RSLC Acclaim PepMap C18 column (150 mm length, 75 µm inner diameter, 1.8 µm particle size, 100 A pore size) using a linear gradient from 98% solvent A (water, 0.08% formic acid, 0.015% trifluoroacetic acid) and 2% solvent B (0.08% formic acid, 0.015% trifluoroacetic acid in acetonitrile) to 26% solvent B over 120 min at a flow rate of 0.3 µL/min, then increasing to 85% of solvent B for 10 min, washing the column in 85% of solvent B for 10 min following column equilibration at initial conditions of eluting gradient for the next 15 min. The total analysis time was 155 min.

Raw data files were converted to fit-for-searching format using MSConvert (Proteome Wizard). Protein identification was performed using MASCOT software version 2.6 (https://www.matrixscience.com (accessed on 1 October 2025)). All tandem mass spectra were searched against the customized proteins database originated from transcripome analysis. The following search parameters were used: Trypsin was used as the cutting enzyme and up to two missed cleavages were allowed. The mass tolerance window for the monoisotopic precursor ions was set to ±10 ppm, while the mass tolerance window for fragment ions was set to ±0.02 Da. Cysteine pyridilethylation and oxidation of methionine were chosen as variable modifications. The criteria for positive identification were set as follows: minimum score of 50, at least three positive identifications from three different runs. Final results were extracted at no more than 1% of FDR level based on the summarized false discovery rate for PSMs, peptides and proteins with dynamic correction of the recovered sequence with the molecular weight of the canonical protein sequence.

The mass spectrometry measurements were performed using the equipment of “Human Proteome” Core Facilities of the Institute of Biomedical Chemistry (Moscow, Russia).

### 2.7. Estimating of Protein Content in the Proteome

To determine the normalized relative amount of protein content in the proteome, the exponentially modified Protein Abundance Index (emPAI), developed for evaluating LC-MS/MS analysis data, was used [47]. The proteins abundance was determined using the formula:

emPAI = 10^PAI^ − 1, with PAI = N_observed peptides_/N_observable peptides_

The emPAI values were extracted for each protein within the studied groups, and data with zero means were imputed. A measure of protein abundance emPAI was normalized to the housekeeping protein glyceraldehyde-3-phosphate dehydrogenase (GAPDH, KFH47131.1) and represented as a median value fold changes (FC) ratio toward the control group. The number of «observable» peptides per protein was calculated by the Protein Digestion Simulator program (PNNL, current release 2.4.8937). To facilitate visualization and comparison, samples with missing emPAI values for a particular protein were assigned half the minimum emPAI value for that protein in the data set.

### 2.8. Data Analysis

Hierarchical cluster analysis of differentially expressed proteins (DEPs) was calculated in R v.4.2.1 programming language using hclust function. Principal components analysis (PCA) was performed in R programming language using prcomp function.

Statistically overrepresented GO terms in DEPs were identified using clusterProfiler v4.6.2 R package [48], GO terms with adjusted *p*-value ≤ 0.05 was considered significantly enriched. *p*-values were adjusted using Benjamini–Hochberg procedure, GO annotations from UniProt database were used [49].

## 3. Results

Proteomic analysis was performed for samples collected after 72, 96, and 120 h of submerged fermentation of *A. chrysogenum* HY and WT strains on CP complex medium using liquid chromatography tandem mass spectrometry (LC-MS/MS). Time points were selected on the basis of previous experimental data. During fermentation of *A. chrysogenum* HY on CP medium, after 72 h, there was a sharp increase in the production of cephalosporin C, up to 5500–6000 mg/L; by the end of fermentation (120 h), the yield was more than 10,000 mg/L [9,23,31]. In the WT strain, throughout the entire fermentation period under these conditions, the production of cephalosporin C did not exceed 30–50 mg/L [7]. This made it possible to study the changes in the proteome of *A. chrysogenum* HY during the period of active cephalosporin C biosynthesis, as well as to compare the protein composition of the two strains at these time points.

### 3.1. Alterations in the Proteome of A. chrysogenum HY Compared to A. chrysogenum WT at Different Time Points Fermentation Time Points

**Figure 1:**
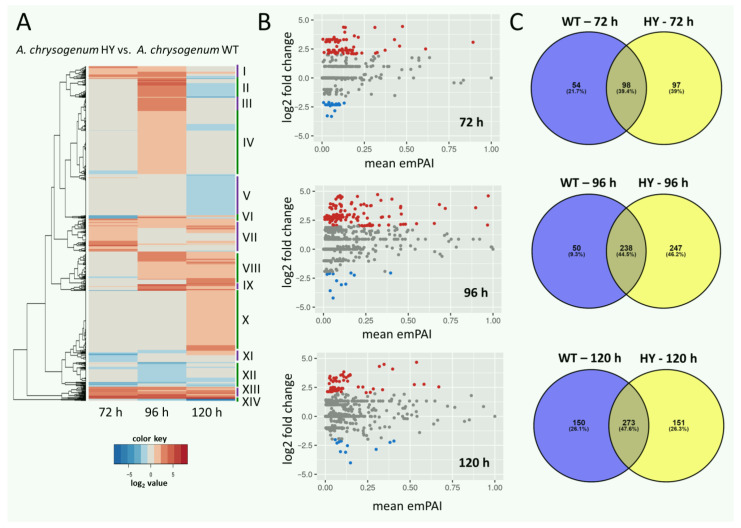
Differentially expressed proteins (DEPs) in *A. chrysogenum* WT and HY strains after submerged fermentation on complex CP medium for 72, 96, and 120 h. (**A**)—Hierarchical cluster analysis; log2 fold change in expression values are shown as a color scale, with upregulated DE proteins represented by red and downregulated DE proteins represented by blue. Clusters with similar differential expression trends during fermentation are numbered I–XIV. (**B**)—MA plots for comparative analysis of protein production in *A. chrysogenum* HY versus WT. The M parameter was defined as log2 (HY_emPAY_/WT_emPAY_). Gray dots represent proteins with similar levels of regulation, red dots represent proteins with significantly increased regulation, blue dots represent proteins with significantly decreased regulation; significance threshold was absolute log2 fold change ≥ 1 and adjusted *p*-value ≤ 0.05. (**C**)—Venn diagrams for comparison of proteins of two strains isolated at different times of fermentation.

To determine the changes that occurred in the CSI program, a comparative analysis of the proteomes of *A. chrysogenum* WT and HY strains cultured for the same period of time was performed (Figure 1, Tables S1 and S2). A significant number of proteins were found to be differentially expressed. After 72 h, 39.4% of DEPs were detected, and the value increased to 44.5% at 96 h and 47.6% to the end of fermentation (Figure 1C).

Hierarchical cluster analysis showed that there are groups of proteins that have similar trends in differential expression (HY/WT) during fermentation (Figure 1A, Table S3). Clusters were identified for which both upregulation of HY proteins and downregulation, or a change in upregulation and downregulation, were observed throughout the fermentation process. The strongest upregulation of proteins of the HY strain throughout the entire fermentation period (10–100 times) was observed in cluster XIII. In cluster I, strong upregulation (3–15 times) was characteristic up to 96 h; at the end of the fermentation period, it significantly decreased. In cluster VII, significant regulation was observed at the beginning of fermentation, and then, it decreased for most proteins in the cluster. In cluster X, no significant change in the level of DEPs was observed at the first stages of fermentation; after 120 h, there was a pronounced upregulation. Downregulation was observed in clusters: V (at the beginning of fermentation there were no significant differences, after 120 h of fermentation downregulation was 3–5 times), XII (the strongest upregulation was for 96 h), and a small cluster XIV (for which an increase in downregulation was observed throughout fermentation; after 120 h, downregulation occurred 10–100 times). In the remaining cluster, there was no clear trend. For example, the relative expression of proteins in cluster II was not detected at 72 h; however, it was significantly upregulated (5- to 10-fold or more) at 96 h, before being downregulated again by 120 h (up to 10 times). MA plot analysis showed that during the entire fermentation period, the number of unregulated proteins in *A. chrysogenum* HY exceeded the number of downregulated ones (Figure 1B). This ratio is most pronounced after 96 h of fermentation.

**Figure 2:**
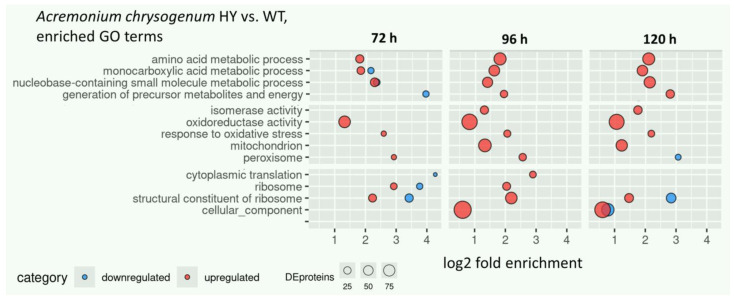
Enrichment analysis of overrepresented GO terms in DEPs in *A. chrysogenum* strains (HY vs. WT) after submerged fermentation on complex CP medium for 72 h, 96 h, and 120 h. Only terms with adjusted *p*-value ≤ 0.05 are displayed.

GO enrichment analysis showed that the fold enrichment of GO terms in *A. chrysogenum* (HY vs. WT) increased after 96 h of fermentation. The proportion of DEP increased after 96 h compared to 72 h. Thus, as a consequence, the fold enrichment of GO terms (circle size) increased after 96 h (Figure 2, Table S4). Furthermore, the total number of statistically enriched GO terms in upregulated DE proteins exceeded the number of downregulated ones. The amino acid metabolic process is overrepresented in *A. chrysogenum* HY throughout the analyzed fermentation stages (Figure 2). Such changes in the HY strain may lead to increased production of primary building blocks and energy sources. Overrepresentation of this process was previously observed in a comparative transcriptome analysis of improved beta-lactam producers *A. chrysogenum* and *P. chrysogenum* with the corresponding wild-type parental strains [50]. For monocarboxylic acid metabolic and nucleobase-containing small molecule metabolic processes, overrepresentation in both upregulated and downregulated proteins was observed after 72 h. At subsequent stages of fermentation, changes in these GO terms were associated only with upregulation. The term GO generation of precursor metabolites and energy was enriched in downregulation after 72 h of fermentation, then enriched in upregulated proteins until the end of fermentation. Such changes may also indicate a comparative intensification of the metabolism of *A. chrysogenum* HY in the late stages of fermentation, when active production of cephalosporin C occurs.

In *A. chrysogenum* HY, upregulation was also found for the GO terms: isomerase activity, oxidoreductase activity, response to oxidative stress, and mitochondrion at all stages of fermentation. For peroxisome, upregulation occurred for up to 96 h and downregulation was observed after 120 h (Figure 2). For GO terms such as cytoplasmic translation, ribosome, structural constituent of ribosome, and cellular component, upregulation was observed for the 96 h proteomes, with both upregulation and downregulation occurring at other time points.

In *A. chrysogenum* HY, enrichment in upregulated proteins was also found for the following GO terms: isomerase activity, oxidoreductase activity, response to oxidative stress, and mitochondrion at all stages of fermentation; for peroxisome, overrepresentation occurred in upregulation for up to 96 h inclusive and in downregulation it was observed after 120 h (Figure 2). For GO terms such as cytoplasmic translation, ribosome, structural constituent of ribosome, and cellular component, overrepresentation in upregulation is observed for the 96 h proteomes, with statistical enrichment both occurring during upregulation and downregulation at other time points.

The obtained comparative proteomic data indicate significant changes that occurred in the *A. chrysogenum* HY strain in the SCI program as a whole. These changes show a general trend but vary depending on the fermentation stage at which the comparisons were made. This may be due to the fact that different groups of proteins are produced at different fermentation periods.

### 3.2. Proteome Rearrangement in A. chrysogenum WT and HY Strains During Fermentation

**Figure 3:**
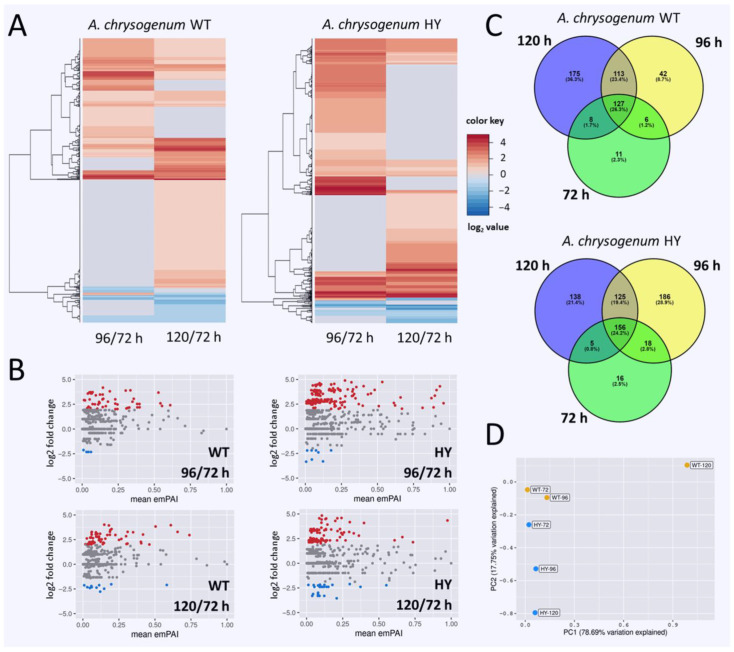
Proteome rearrangement in *A. chrysogenum* WT and HY strains during fermentation. DEPs, isolated from *A. chrysogenum* WT (96 vs. 72 h and 120 vs. 72 h) and HY (96 vs. 72 h and 120 vs. 72 h): (**A**)—hierarchical cluster analysis, expression values are shown as a color scale, with higher values represented by red and lower represented by blue; (**B**)—MA plots, the M parameter defined as log2(WT96_emPAY_/WT72_emPAY_), or log2(WT120_emPAY_/WT72_emPAY_), log2(HY96_emPAY_/HY72_emPAY_), or log2(HY120_emPAY_/HY72_emPAY_). Gray dots represent proteins with similar levels of regulation, red dots represent proteins with significantly increased regulation, blue dots represent proteins with significantly decreased regulation; significance threshold was absolute log2 fold change ≥ 1 and adjusted *p*-value ≤ 0.05. (**C**)—Venn diagrams for DEPs from WT and HY, isolated after 72, 96, and 120 h of fermentation. (**D**)—Principal component analysis for proteomes of *A. chrysogenum* strains (WT-72, WT-96, WT-120, HY-72, HY-96, and HY-120).

To determine the impact of fermentation stages on differential protein expression, we also analyzed the changes that occurred in individual *A. chrysogenum* strains, WT and HY, during fermentation. Comparative analysis of the proteomes of *A. chrysogenum* WT isolated after 72, 96, and 120 h of fermentation and the proteomes of *A. chrysogenum* HY isolated at the same time points revealed significant rearrangements for both strains, especially at the late stages of fermentation. After 72 h in the WT and HY strains 2.3% and 2.5% of proteins were differentially expressed, respectively; by 96 h, these values increase to 8.7% and 28.9%, respectively (Figure 3, Tables S1 and S2). The 28.9% increase in differential expression in *A. chrysogenum* HY after 96 h may indicate proteome reorganization to produce high-yielding products, which is enhanced during this stage and continues for 120 h (21.4% DEP) [9]. After 120 h, *A. chrysogenum* WT shows a sharp increase in the amount of DEPs, up to 36.3%, which may indicate a transition to the stage of conidia formation, which is typical for this strain at the end of fermentation [10].

Hierarchical cluster analysis and MA plots showed that, compared to 72 h, at later stages of fermentation, in both strains, the number of upregulated proteins exceeded the number of downregulated proteins against the background of a significant number of proteins that showed no changes in production level (Figure 3A,B; Table S5). Moreover, when comparing 96/72 h and 120/72 h, upregulation and downregulation may involve different groups of proteins in both strains. In *A. chrysogenum* HY, overall, there is a stronger differential expression, with a cluster of proteins, which are heterogeneous in their functions, upregulated 10–20 times at both time points compared to that observed at 72 h (Figure 3A, Table S5).

Principal component analysis showed that the proteomes of *A. chrysogenum* WT obtained after 72 and 96 h of fermentation (WT-72 and WT-96) were relatively similar (Figure 3D). The proteome of *A. chrysogenum* HY-72 is also localized near them. The HY-96 proteome is relatively distant from HY-72. The proteomes of both strains obtained after 120 h of fermentation (WT-120 and HY-120) are the most distant both from the previous proteomes of these same strains and from each other. Such rearrangement within the proteomes of both strains indicates significant changes occurring at the final stage of fermentation. Moreover, in the wild-type strain these changes are abrupt; the proteomes after 72 and 96 h are close, while sharp differences appear after 120 h. For the HY strain, proteome changes during fermentation occur sequentially: the HY-96 proteome moves away from HY-72 by approximately the same distance as the HY-120 proteome moves away from HY-96 (Figure 3D).

**Figure 4:**
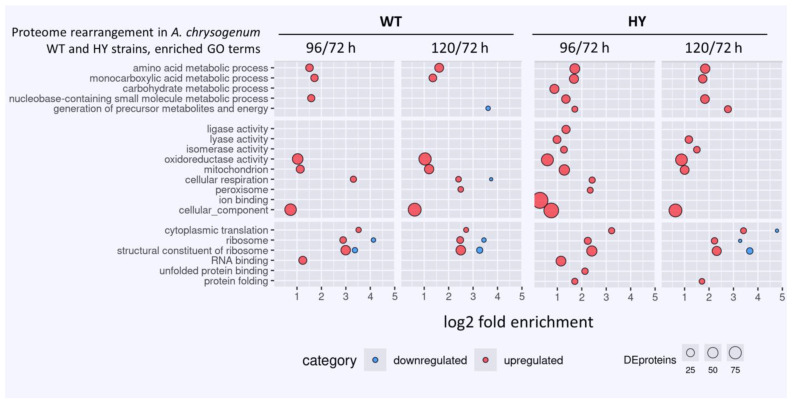
Enrichment analysis of overrepresented GO terms in DEPs in *A. chrysogenum* strain WT and HY during fermentation, 96 vs. 72 h and 120 vs. 72 h. Only terms with adjusted *p*-value ≤ 0.05 are displayed.

GO enrichment analysis also demonstrates a predominance of upregulated GO terms compared to downregulated ones. (Figure 4, Table S6). The bubble map shows similar trends both when comparing WT 96/72 h with WT 120/72 h and HY 96/72 h with HY 120/72 h. An important difference is revealed for the “generation of precursor metabolites and energy.” While the WT strain showed no changes for this GO term at 96/72 h and downregulation at 120/72 h, upregulation occurs in HY 96/72 h and HY 120/72 h, which may indicate the mobilization of cellular resources for high-yield antibiotic production. In the *A. chrysogenum* HY strain, upregulation of GO terms such as carbohydrate metabolic process, ligase activity, lyase activity, isomerase activity, ion binding, unfolded protein binding, and protein folding were also detected. In the WT strain, no changes in these GO terms occurred when compared with 72 h of fermentation.

The obtained data indicate that after 72 h of fermentation, both *A. chrysogenum* strains undergo processes that lead to significant rearrangements of the proteomes. The increased upregulated GO terms in *A. chrysogenum* HY compared to WT strains may indicate that this strain more actively restructures the proteome for high-yield production in the late fermentation period (Figure 4). The most important specific alterations are discussed in the following sections.

### 3.3. Upregulation of Enzymes of the CPC Biosynthesis Pathway in A. chrysogenum HY

**Figure 5:**
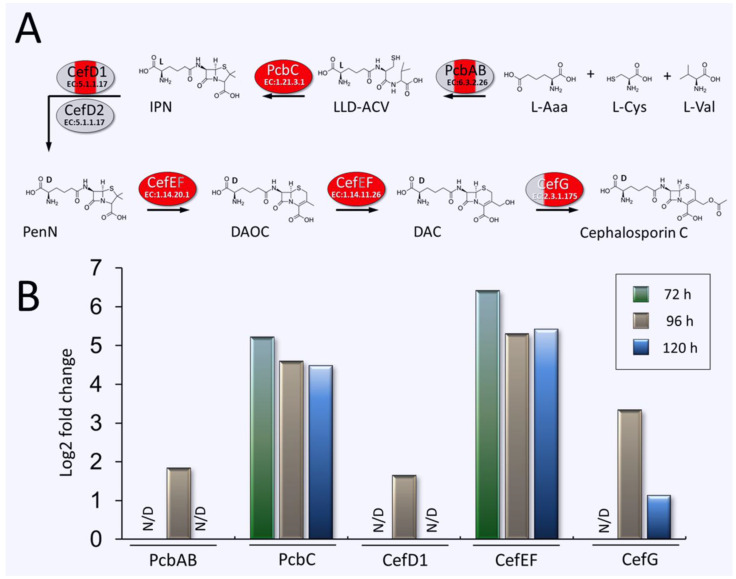
Upregulation of cephalosporin C biosynthetic enzymes in *A. chrysogenum* HY strain relative to WT. (**A**)—beta-lactam biosynthesis pathway in *Acremonium chrysogenum*. Enzymes for the “early” stages of beta-lactam biosynthesis: PcbAB—ACV (δ-[L-α-Aminoadipyl]-L-Cysteinyl-D-Valine) synthetase (EC: 6.3.2.26), PcbC—isopenicillin N-synthase (EC: 1.21.3.1); “middle” stages: CefD1—isopenicillin N-CoA synthetase (EC: 5.1.1.17), CefD2—isopenicillin N-CoA epimerase (EC: 5.1.1.17); enzymes for the “late”: CefEF—deacetoxycephalosporin C synthetase (penicillin N expandase, EC: 1.14.20.1)/deacetoxycephalosporin C hydroxylase (EC: 1.14.11.26), CefG—deacetylcephalosporin-C acetyltransferase (EC: 2.3.1.175). L-Aaa—L-α-aminoadipic acid; LLD-ACV—δ-(L-α-aminoadipoyl)-L-cysteinyl-D-valine; IPN—isopenicillin N; PenN—penicillin N; DAOC—deacetoxycephalosporin C; DAC—deacetylcephalosporin C. Upregulated proteins in *A. chrysogenum* HY filled in red; proteins for which no distinctions were made filled in gray. Initial one third of the fill corresponds to 72 h of fermentation, the middle third, 96 h, and the last third, 120 h. (**B**)—Differential expression levels log2 (HY_emPAI_/WT_emPAI_) of enzymes PcbAB, PcbC, CefD1, CefEF and CefG after 72, 96 and 120 h of fermentation. N/D—not detected.

In *A. chrysogenum*, CPC biosynthesis occurs due to the sequential functioning of six biosynthetic enzymes: PcbAB (KFH48607.1, EC: 6.3.2.26), PcbC (KFH48817.1, EC: 1.21.3.1), CefD1 (KFH48713.1, EC: 5.1.1.17), CefD2 (KFH48581.1, EC: 5.1.1.17), CefEF (KFH44919.1, EC: 1.14.20.1)/EC: 1.14.11.26) and CefG (KFH44925.1, EC: 2.3.1.175) (Figure 5A). In HY the strain, upregulation of all these enzymes was detected at different stages of fermentation, with the exception of CefD2, for which no differences were found and the weakest mRNA upregulation, among CPC biosynthetic enzymes, was previously shown [7] (Figure 5). The upregulation was quite strong and in some cases reached 70–80 times; downregulation was not observed at any stage of *A. chrysogenum* HY fermentation.

PcbAB, a 3-module NRPS for constructing the core structure of beta-lactams, was upregulated after 96 h by approximately 4 folds. Among the tailoring enzymes, PcbC and CefEF were particularly strongly upregulated, increasing by 20–80 times throughout the observed fermentation period. CefD1 was upregulated approximately 3 times after 96 h; CefG was upregulated more than 10 times after 96 h and approximately 2 times after 120 h. In absolute terms, PcbC and CefEF have fairly high emPAI values, around 1, which corresponds to 1–3% of all proteins in the proteome of *A. chrysogenum* HY (Table S2). After 96 h, the content of CefG was approximately 10 times lower in the HY strain; the content of PcbAB and CefD1 was even lower: 2–3 times.

### 3.4. Upregulation of Oxidative Stress Proteins in A. chrysogenum HY

**Figure 6:**
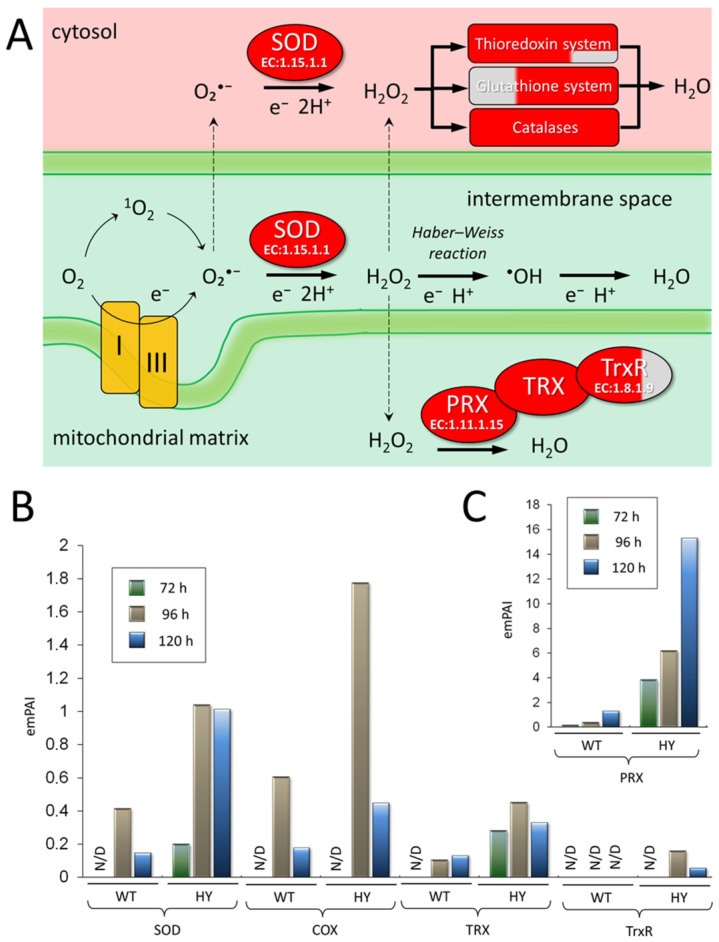
Oxidative stress response in *A. chrysogenum* WT and HY. (**A**)—Schematic representation of reactive oxygen species metabolism in *A. chrysogenum* strains. Upregulated enzymes in (**A**). chrysogenum HY filled in red, which no distinctions filled in gray. Initial one third of the fill corresponds to 72 h of fermentation, the middle third,—96 h, and the last third,—120 h. (**B**,**C**)—The production level of oxidative stress proteins in *A. chrysogenum* WT and HY strains after 72, 96 and 120 h of fermentation. SOD—superoxide dismutase (EC 1.15.1.1); COX—cytochrome c oxidase (7.1.1.9); TRX—thioredoxin, TrxR–thioredoxin reductase (EC 1.8.1.9); PRX—mitochondrial peroxiredoxin (EC 1.11.1.15). N/D—not detected.

**Figure 7:**
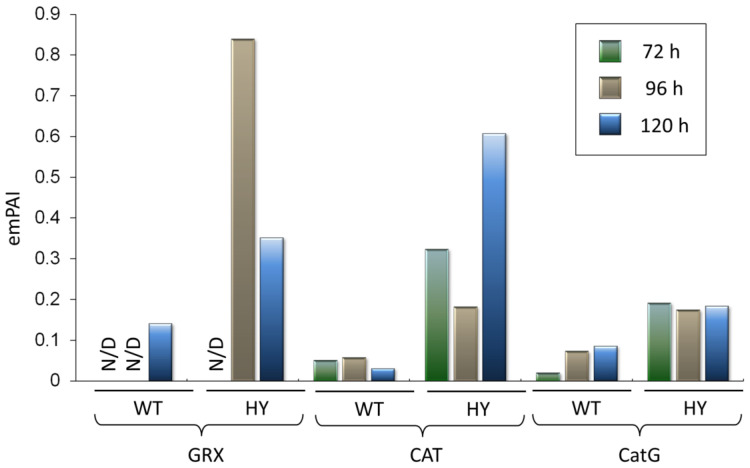
The production level of oxidative stress proteins in *A. chrysogenum* WT and HY strains after 72, 96 and 120 h of fermentation. GRX—glutaredoxin; CAT—catalase (EC 1.11.1.6), CatG—catalase-peroxidase (EC 1.11.1.21). N/D—not detected.

*A. chrysogenum* HY showed increased production of various components of the oxidative stress system involved in the metabolism of reactive oxygen species (ROS) [51,52] (Figure 6). Superoxide anion radical (O_2_•^−^) is formed when a normal molecular oxygen (O_2_) molecule acquires an electron, which triggers a cascade of reactions associated with the conversion of ROS (Figure 6A) [53]. The main sources of O_2_•^−^ formation are complexes I and III of the mitochondrial respiratory chain [52,54,55] (Figure 6A). At the first stage, superoxide dismutase (SOD, KFH41747.1, EC 1.15.1.1) catalyzes the formation of molecular oxygen and hydrogen peroxide (H_2_O_2_) from two O_2_•^−^ molecules [56,57,58]. In *A. chrysogenum* HY, the superoxide dismutase [Cu-Zn] content is 4 times higher after 96 h of fermentation and 10 times higher after 120 h (Figure 6B). Then in the fungal cell, the hydrogen peroxide generated during the dismutase activity of SOD is utilized by: (i) the thioredoxin system, represented by thioredoxin (TRX), peroxiredoxin (PRX, EC 1.11.1.15), and thioredoxin reductase (TrxR, EC 1.8.1.9); (ii) the glutathione system, represented by glutathione (GSH), glutathione reductase (GR, EC 1.8.1.7), glutathione peroxidase (GPx EC 1.11.1.9), and glutaredoxin (GRX) [59]; (iii) catalase (CAT, EC 1.11.1.6) (Figure 6A) [60]. We found a significant increase in the content of the main components of these systems in *A. chrysogenum* HY (Figure 6 and Figure 7).

In the thioredoxin system, PRX is oxidized in the reaction of hydrogen peroxide reduction to water. PRX is then reduced by TRX, which in turn is oxidized and reduced by TrxR using NADPH [61,62]. PRXs are known to be extremely abund ant proteins, accounting for approximately 1% of soluble proteins in the proteome [63,64]. These proteins are also extremely effective in neutralizing peroxides. PRXs are thought to act as redox sensors that transmit signals in response to oxidative stress, including in the mitochondria, the main source of hydrogen peroxide [54]. In this regard, PRXs will play a central role in the response of cells to oxidative stress and redox signaling processes [65]. The amount of mitochondrial peroxiredoxin (PRX, KFH47331.1, EC 1.11.1.15) consistently increased during the fermentation of both strains and was 15–25 times higher in the HY strain (Figure 6C). Consequently, the PRX content was the highest among the proteins of the *A. chrysogenum* HY, reaching 3.87% after 72 h, 6.2% after 96 h, and 15.32% after 120 h of the proteome composition (Table S2).

The thioredoxin (TRX, KFH42608.1) content in *A. chrysogenum* HY was increased 2–3 times after 96 and 120 h of fermentation and was detectable after 72 h, in contrast to the findings for the WT strain (Figure 6B). Thioredoxin reductase (TrxR, KFH40674.1, EC 1.8.1.9) was detected only in *A. chrysogenum* HY proteomes isolated after 96 h of fermentation.

We also noted an increase in the content of cytochrome-c-oxidase (COX, KFH48595.1, EC 7.1.1.9), the rate-limiting and terminal protein complex of the mitochondrial respiratory chain. COX content in *A. chrysogenum* HY was 3 times higher after 96 h and 120 h (Figure 5B). This increase may have been due to the previously described phenomenon of mitochondrial biogenesis and an increase in their content under conditions of enhanced ROS production [66]. Currently, there is no clear understanding of the relationship between COX function and reactive oxygen species, but there are models suggesting that these stress factors may have an indirect effect on increasing the cytosolic calcium concentration, which leads to mitochondrial activation from the “resting state” [67].

Glutaredoxin (GSH, KFH42494.1) upregulation was also observed in the late stages of fermentation. After 72 h, this component of the glucoredoxin system for hydrogen peroxide utilization was not observed in either strain (Figure 7). In *A. chrysogenum* HY, catalase (CAT, KFH49103.1, EC 1.11.1.6) content, an important enzyme regulating oxidative stress [68,69], was 3–6 times higher after 72–96 h and 20 times higher after 120 h (Figure 7). Another enzyme capable of utilizing hydrogen peroxide into water, catalase-peroxidase (CatG, KFH44281.1, EC 1.11.1.21), differs from peroxidase (EC 1.11.1.7) in its relatively high catalase activity (EC 1.11.1.6) due to its unique mechanism of catalysis [70,71,72]. The CatG content increased in *A. chrysogenum* HY by 5–10 times after 72 h and by 2–3 times at subsequent stages of fermentation. After 120 h of fermentation, *A. chrysogenum* HY also showed increased levels of the enzyme glutathione S-transferase (GST, KFH40871.1, 2.5.1.18) (Table S2). This change may also be a response to oxidative stress in *A. chrysogenum* HY, as glutathione S-transferase has been shown to also protect cells from oxidative stress as it exhibits GSH-dependent peroxidase activity [59,73].

The underlying reasons of upregulation of oxidative stress proteins are discussed in detail in Section 4, with visual representations provided in corresponding Figures. These figures illustrate the connection between oxidative stress in *A. chrysogenum* HY and other crucial alterations required for the efficient production of cephalosporin C.

### 3.5. Upregulation of Sulfur Metabolism Enzymes in A. chrysogenum HY

**Figure 8:**
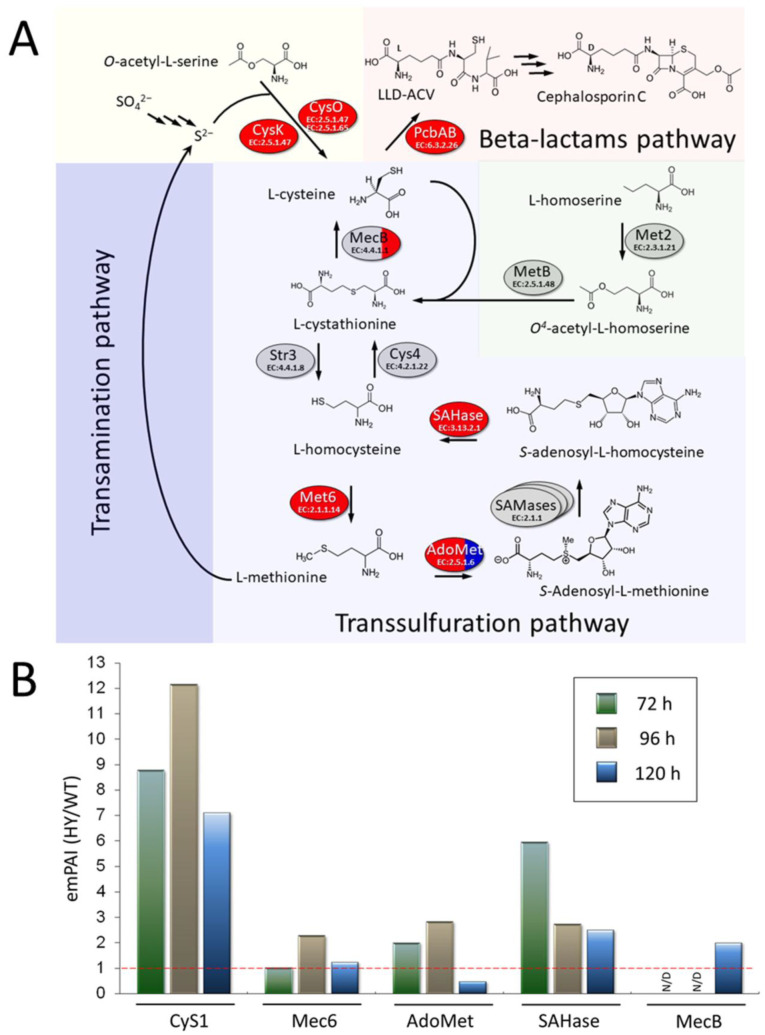
Sulfur metabolism in *A. chrysogenum* WT and HY. (**A**)—Changes in sulfur metabolism in *A. chrysogenum* HY. Enzymes shaded in red are those for which upregulation was observed in *A. chrysogenum* HY compared to WT at all stages of fermentation. AdoMet, which showed upregulation up to 96 h and downregulation after 120 h, is shaded in red to blue. Enzymes for which no distinctions were made are shaded in gray. CyS—cysteine synthase (EC 2.5.1.47); CysO—cysteine synthase 2 (EC 2.5.1.65); MecB—cystathionine gamma-lyase (EC 4.4.1.1); Str3—cystathionine beta-lyase (EC 4.4.1.8); Met6–5-methyltetrahydropteroyltriglutamate-homocysteine *S*-methyltransferase (EC 2.1.1.14); AdoMet—S-adenosylmethionine synthase (EC 2.5.1.6); SAMase—SAM-dependent methylases (EC 2.1.1); SAHase—Adenosylhomocysteinase (EC 3.13.2.1); Cys4—cystathionine beta-synthase (EC 4.2.1.22); Met2—homoserine O-acetyltransferase (EC 2. 3.1.21); MetB—cystathionine gamma-synthase (EC 2.5.1.48). (**B**)—Differential expression levels (HY_emPAI_/WT_emPAI_) of sulfur metabolism proteins. The dotted line shows the level at which there are no differences; N/D—not detected.

Sulfur metabolism is critical for beta-lactam biosynthesis because one of the three amino acids required for synthesis, cysteine, contains a sulfur residue (Figure 5) [74]. Cysteine can be produced by the activities of cysteine synthases CysK (1298 AA, KFH49003.1, EC 2.5.1.47 and CysO (443 AA, KFH44701, EC 2.5.1.65) from *O*-acetylserine and hydrogen sulfide or cystathionine gamma-lyase (MecB, EC 4.4.1.1) from cystathionine (Figure 8). CyS utilizes inorganic sulfur in the form of hydrogen sulfide, obtained from the assimilatory sulfate reduction pathway; MecB catalyzes the final reaction in the methionine transsulfuration pathway (Figure 8). Hydrogen sulfide for the incorporation of sulfur into cysteine can also be obtained from the catabolism of methionine (Figure 8).

Our experiments showed that in *A. chrysogenum* HY, some key enzymes of sulfur metabolism, in particular cysteine biosynthesis, are upregulated. The greatest increase in the content, by 7–12 times, occurred in CysK; upregulation was observed at all stages of fermentation (Figure 8B, Table S3). Also, after 120 h, upregulation was observed for enzymes of the assimilatory sulfate reduction pathway, such as adenylyl-sulfate kinase (CysC, KFH42682.1, EC 2.7.1.25) and sulfite reductase (CysJ, KFH42074.1, EC:1.8.1.2). Such alterations could lead to the efficient consumption of inorganic sulfur supplied in the form of sulfates and its incorporation into the cysteine molecule.

Also, at all stages of *A. chrysogenum* HY cultivation, the production of enzymes of the transsulfarylation pathway increased, such as MecB-cystathionine gamma-lyase (KFH41360.1, EC 4.4.1.1) (by, up to 2 times); Met6–5-methyltetrahydropteroyltriglutamate-homocysteine S-methyltransferase (KFH43460.1, EC 2.1.1.14) (by, up to 2 times); and SAHase–Adenosylhomocysteinase (KFH42402.1, EC 3.13.2.1) (by 3–6 times) (Figure 8B). For AdoMet–S-adenosylmethionine synthase (KFH48080.1, EC 2.5.1.6), upregulation was observed up to 96 h inclusive, by 2–3 times; after 120 h downregulation was observed, by 2 times. The data obtained indicate an intensification of the transsulfuration pathway as a whole, which can have a positive effect both on the production of “building blocks”, cysteine molecules, and on the construction of methionine, which plays a key role in metabolism and growth control in fungi [75]. Also, upregulation of the transmethylation pathway (including Met6, AdoMet, and SAHase) at most stages of fermentation may positively impact the biosynthesis of S-adenosylmethionine (SAMe), a key methyl donor in the epigenetic regulation of secondary metabolic pathways [76,77,78,79].

### 3.6. Upregulation of Selected Enzymes of Primary Metabolism in A. chrysogenum HY

**Figure 9:**
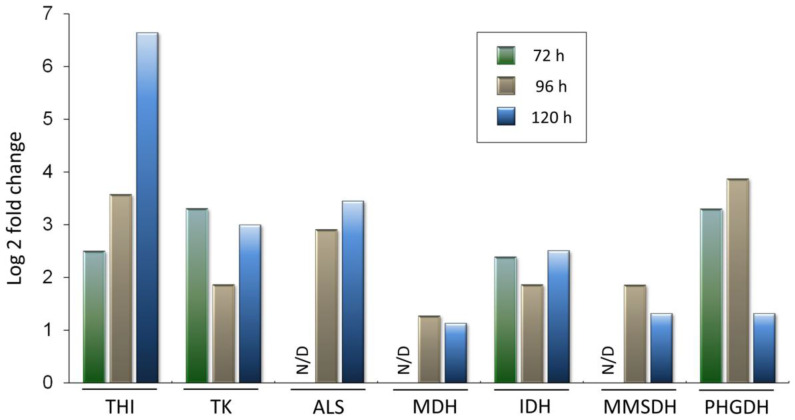
Upregulation of selected primary metabolism enzymes in *A. chrysogenum* HY. Differential expression levels log2 (HY_emPAI_/WT_emPAI_) of enzymes: THI—thiamine thiazole synthase (EC: 2.4.2.60); TK—transketolase (EC: 2.2.1.1); ALS—acetolactate synthase (EC: 2.2.1.6); MDH—malate dehydrogenase (EC: 1.1.1.37); IDH—isocitrate dehydrogenase (EC 1.1.1.41); MMSDH—methylmalonate-semialdehyde dehydrogenase (EC: 1.2.1.27); PHGDH—phosphoglycerate dehydrogenase (D-3-phosphoglycerate dehydrogenase, EC: 1.1.1.95). N/D—not detected.

Thiamine thiazole synthase (THI, KFH43147.1, EC: 2.4.2.60) turned out to be one of the most abundant enzymes in the *A. chrysogenum* HY proteomes at all stages of fermentation (Figure 9). It was overexpressed 6-fold after 72 h, 13-fold after 96 h, and approximately 100-fold after 120 h when compared with the WT strain. Such upregulation can be due to the increased demand for its product, a cofactor of a number of key enzymes, thiamine pyrophosphate (TPP) and the suicidal nature of this single-turnover enzyme THI, which is inactivated during the catalytic reaction [80,81]. Significant upregulation of THI was shown previously for the *A. chrysogenum* strain 84-3-81-41[41].

A number of enzymes that use the TPP cofactor, such as transketolase (TK, KFH45245.1, EC: 2.2.1.1) and acetolactate synthase (ALS, KFH44601.1 EC: 2.2.1.6), are also overexpressed. TK is an important enzyme that allows excess sugars to be removed from the pentose phosphate pathway into glycolysis, with subsequent energy production [82]. During fermentation, TK is upregulated by 4–10 times in *A. chrysogenum* HY during (Figure 9). ALS catalyzes the first reaction in the biosynthetic pathway of valine (and other branched-chain amino acids such as leucine and isoleucine) from pyruvate [83]. Valine is one of three amino acids required for the synthesis of beta-lactams (Figure 5). Therefore, upregulation of the enzyme responsible for its biosynthesis, by 8–10 times, is seen as a positive factor for high-yielding production.

Also, in *A. chrysogenum* HY, some enzymes of the tricarboxylic acid cycle are upregulated, such as malate dehydrogenase (MDH, KFH43115.1, EC: 1.1.1.37) and isocitrate dehydrogenase (IDH, KFH45057.1 EC 1.1.1.41). MDH was upregulated by 2–2.5 times after 96 and 120 h. For IDH, a stronger upregulation was observed, 4–5 times throughout the fermentation. Importantly, along with the upregulation of IDH, which synthesizes α-ketoglutarate (2-oxoglutarate) from isocitrate, there was a downregulation of the enzymatic complex that metabolizes α-ketoglutarate to succinyl-CoA (Table S2). These changes are possibly related to the consumption of α-ketoglutarate as a substrate for the conversion of PenN to DAOC by the CefEF enzyme of the CPC biosynthetic pathway. More details on these reactions are provided in the Discussion section.

Phosphoglycerate dehydrogenase (D-3-phosphoglycerate dehydrogenase, PHGDH, KFH46270.1, EC: 1.1.1.95) catalyzes a reaction in the serine biosynthetic pathway, which is essential in cysteine synthesis. This enzyme is upregulated in *A. chrysogenum* HY more than 10-fold after 72 and 96 h of fermentation and 2–3-fold after 120 h (Figure 9). Methylmalonate-semialdehyde dehydrogenase (MMSDH, KFH42979.1, EC: 1.2.1.27) is involved in the metabolic pathways of inositol and propanoate, as well as in the degradation of branched-chain amino acids, valine, leucine and isoleucine. With active consumption of valine for CPC biosynthesis, the observed upregulation of this enzyme, 2–3 times, after 96 and 120 h of fermentation, may be directed towards the catabolism of leucine and isoleucine.

### 3.7. Changes in Grg Protein Production

**Figure 10:**
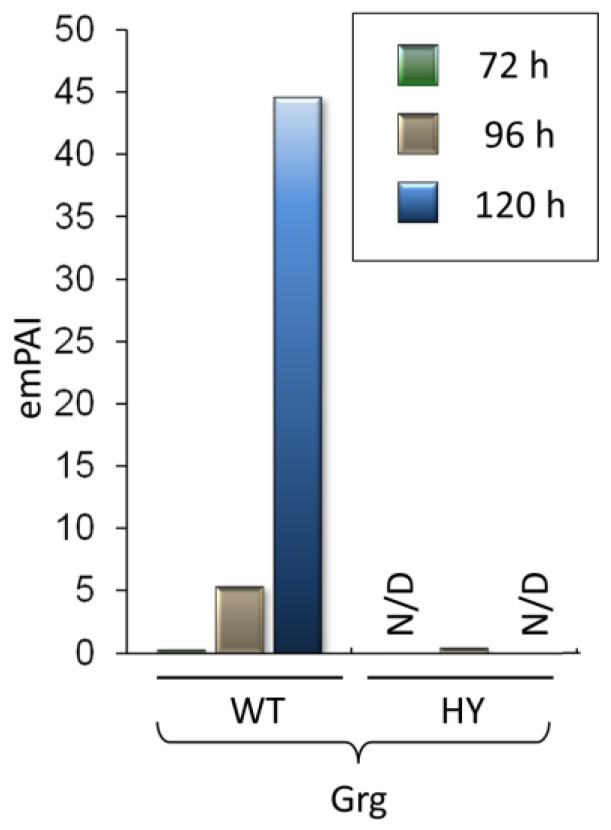
The production level of protein Grg (glucose-repressible gene) in *A. chrysogenum* WT and HY strains after 72, 96 and 120 h of fermentation. N/D—not detected.

In the wild-type strain, the content of the KFH46345.1 protein, called the glucose-repressible gene (Grg), increases significantly during fermentation. After 72 h, its concentration is 1.25% of the total protein content; after 96 h it reaches 8.3%; and after 120 h it accounts for 37.6%, making it the most abundant protein in *A. chrysogenum* WT (Table S2). The emPAI value for Grg in WT-120h reaches 44.6, which is the maximum value for all proteomes analyzed in this work. This protein is strongly downregulated in the HY strain: after 96 h, production is reduced by more than 10 times; after 72 and 120 h, Grg is not detected (Figure 10).

Significant downregulation of this protein in *A. chrysogenum* HY is a positive marker indicating that cells of this strain experience significantly less starvation or demonstrating the recurrence of regulatory mutations that led to an escape from the aging program. Both such events are important for high-yield production of cephalosporin C. The known role of Grg in fungal cell aging and the significance of its downregulation for CPC production in *A. chrysogenum* HY are discussed in more detail in the Discussion section.

## 4. Discussion

**Figure 11:**
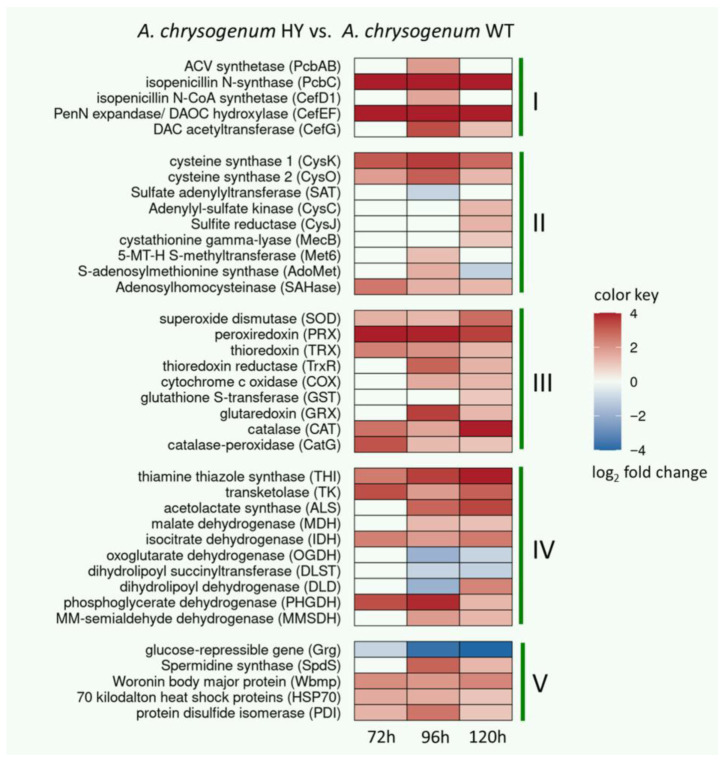
Differentially expressed selected groups of proteins in *A. chrysogenum* WT and HY strains after submerged fermentation on complex CP medium for 72, 96, and 120 h: I—CPC biosynthesis, II—sulfur metabolism, III—oxidative stress, IV—primary metabolism, V—miscellaneous; log2 fold change in expression values are shown as a color scale, with upregulated DE proteins represented by red and downregulated DE proteins represented by blue.

Our work showed that *A. chrysogenum* HY undergoes significant alterations in the composition of the proteome, some of which contribute significantly to high-yield CPC production (Figure 11). Among these, key changes are associated with upregulation of beta-lactam biosynthetic enzymes. The content of enzymes, encoded by genes both from “early” (*pcbAB, pcbC*, *cefD1*) and “late” (*cefEF* and *cefG*) beta-lactam BGCs, in *A. chrysogenum* HY was significantly increased compared that of the parental WT strain (Figure 5, Table S3). These data correlate with the qPCR results, where significant upregulation, 13–370 times, was observed for the corresponding genes *pcbAB*, *pcbC*, *cefD1*, *cefEF* and *cefG* in the HY strain from 48 to 120 h of fermentation [7]. The *cefD2* gene was upregulated 3–7-fold [7], and no differential expression was found for the corresponding CefD2 enzyme in the current study. Among these biosynthetic enzymes, the highest abundance was for PcbC and CefEF. The PcbC content is from proteome proteins: 1.9% after 72 h, 1.2% after 96 h and 0.6% after 120 h; the content of CefEF is 2.8% after 72 h, 1% after 96 h and 0.7% after 120 h (Table S2). Such an increase in the enzyme content could be linked, among other factors, to alterations in the pathway of specific regulation. For instance, it was shown that CefR, regulator, a positive regulator of CefEF [84], is overexpressed in the HY strain [7].

**Figure 12:**
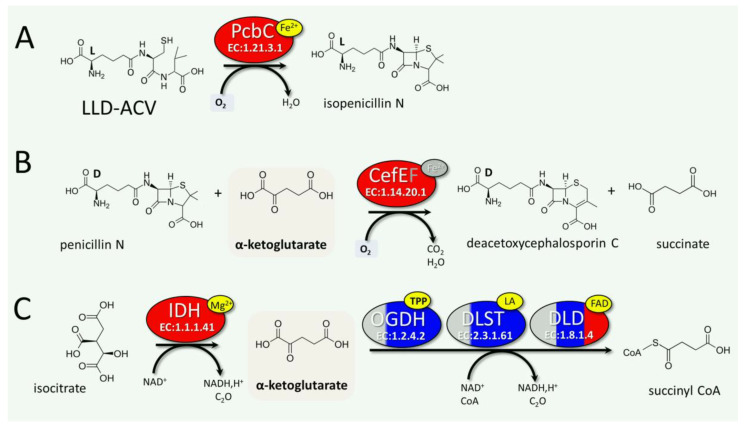
Changes in the content of enzymes catalyzing selected reactions for CPC biosynthesis in *A. chrysogenum* HY. Oxygen-based reactions of the CPC biosynthetic pathway: (**A**)—synthesis of isopenicillin N, (**B**)—synthesis of deacetoxycephalosporin C. Reactions of α-ketoglutarate metabolism: B—consumption in the beta-lactam biosynthesis pathway, (**C**)—biosynthesis and catabolism in the Krebs cycle. LLD-ACV-δ-(L-α-aminoadipoyl)-L-cysteinyl-D-valine; TPP—thiamine phosphate; LA—lipoic acid; FAD—flavin adenine dinucleotide; PcbC—isopenicillin N-synthase (EC: 1.21.3.1); CefEF—deacetoxycephalosporin C synthetase (penicillin N expandase, EC: 1.14.20.1)/deacetoxycephalosporin C hydroxylase (EC:1.14.11.26); IDH—isocitrate dehydrogenase (EC 1.1.1.41); OGDH—oxoglutarate dehydrogenase (EC: 1.2.4.2); DLST—dihydrolipoyl succinyltransferase (EC: 2.3.1.61); DLD—dihydrolipoyl dehydrogenase (EC: 1.8.1.4). Upregulated enzymes in *A. chrysogenum* HY filled in red, downregulated filled in blue, which no distinctions filled in gray. Initial one third of the fill corresponds to 72 h of fermentation, the middle third, 96 h, and the last third, 120 h. Cofactors are filled in yellow, except for the cofactor CefEF, which is involved in the catalysis of the next reaction of this enzyme, not the one shown, and is filled in gray.

Other changes that were detected are thought to contribute to the ability of the CPC biosynthetic pathway to function effectively. PcbC is a dioxeginase that uses an oxygen molecule to cyclize the ACV tripeptide (Figure 12A). Expandase/deacetoxycephalosporin C hydroxylase CefEF in its first reaction uses an oxygen molecule to expand the five-member thiazolidine ring of penG to a 6-membered dihydrothiazine ring using the substrate α-ketoglutarate, also known as 2-oxoglutarate (Figure 12B). Strong aeration is necessary for the efficient supply of PcbC and CefEF with oxygen during biosynthesis [85,86]. Simultaneously, an increase in the oxygen influx into the cell can lead to oxidative stress. This may be one of the reasons for the significant increase in the content of oxidative stress enzymes observed in our study (Figure 6 and Figure 7).

In *A. chrysogenum* HY, redox homeostasis appears to be disrupted by upregulation of the key proteins involved in oxidative stress. Oxidative stress may be associated with high aeration levels in beta-lactam production in improved strains [40,45,87]. Under physiological conditions, ROS act as secondary messengers, but when the balance between ROS production and their removal by the antioxidant system is disturbed, oxidative stress occurs [88]. The increase in the content of antioxidant system components in *A. chrysogenum* HY indicates that due to the high-yield production of cephalosporin C, the amount of ROS exceeds physiological values, and the cell is forced to redirect resources to inactivate their excess amount. In particular, mitochondrial peroxiredoxin was upregulated. This thiol peroxidase functions as part of the mitochondrial reactive oxygen species defense system and plays a key role in cellular defense and oxidative stress perception [52,89]. Peroxiredoxin levels in fungi may serve as a sensor for oxidative stress [90]. In the HY strain, peroxiredoxin levels increase 15–25-fold compared to the WT strain. At all stages of fermentation, this protein is the most abundant protein in the HY proteome. Also, the HY strain has significantly increased content of superoxide dismutase content, which provides the primary stage of ROS scavenging [91].

One of the substrates for the enzymatic reaction in the CPC biosynthetic pathway is α-ketoglutarate (Figure 12B). In addition to participating in the secondary metabolic pathway, it is one of the central ones in primary metabolism, particularly for the tricarboxylic acid cycle (TCA) [92]. This compound is synthesized from isocitrate by the enzyme isocitrate dehydrogenase (IDH, EC 1.1.1.41). Then in TCA, α-ketoglutarate is metabolized to succinyl-CoA by the oxoglutarate dehydrogenase complex, which consists of three enzymes: oxoglutarate dehydrogenase (OGDH, KFH44228.1, EC: 1.2.4.2), dihydrolipoyl succinyltransferase (DLST, KFH42921.1, EC: 2.3.1.61), and dihydrolipoyl dehydrogenase (DLD, KFH43769.1, EC: 1.8.1.4) (Figure 12C). The obtained data are somewhat unexpected given the significant upregulation (up to 80-fold) of thiamine thiazole synthase (Figure 9), which synthesizes the TPP cofactor for OGDH. Furthermore, one of the enzymes of the oxoglutarate dehydrogenase complex, DLD, is upregulated in the final stage of fermentation. In our study, we found that the enzyme that synthesizes α-ketoglutarate, IDH, is upregulated at all stages of fermentation in *A. chrysogenum* HY. However, most enzymes of the α-ketoglutarate-metabolizing complex are downregulated after 96 h of fermentation. This change may lead to an increase in α-ketoglutarate content for high-yielding production in fungal cell. The succinate formed in the expandase reaction can be further used for the functioning of the Krebs cycle, where a number of later-stage enzymes, such as malate dehydrogenase and isocitrate dehydrogenase, are upregulated.

One of the most important changes observed is the intensification of sulfur metabolism, which is aimed at increasing the production of cysteine (Figure 8). Cysteine is one of three amino acids required for the biosynthesis of beta-lactams [93]. Its production is critical for this secondary metabolic pathway. However, cysteine is toxic to fungal cells, which have tight homeostatic controls that limit its levels. Therefore, increasing cysteine levels for high-yield beta-lactam production is quite a challenging task [94]. For high-yield production of these antibiotics, reprogramming of sulfur flows is necessary for incorporation into cysteine, since sulfur is then removed from metabolism with the final beta-lactams. In the fungal cell, there are two main pathways for obtaining sulfur for cysteine biosynthesis: (i) from the sulfur-containing amino acid methionine via the transsulfuration pathway or (ii) from hydrogen sulfide, obtained after reduction from sulfates or transamination of methionine (Figure 8). In the early 2000s, J. Martín and A. Demain formulated the concept of the “methionine–cephalosporin puzzle of *A. chrysogenum*,” since the role of methionine in stimulation of CPC biosynthesis was unclear. Contradictory data were obtained by two leading scientific groups after knockout of the enzyme cystathionine gamma-lyase (MecB, EC 4.4.1.1), which synthesizes cysteine at the last step of the transsulfuration pathway from methionine [95,96]. Our experiments showed that a number of enzymes of the transsulfuration pathway are upregulated, including MecB, but the greatest upregulation (10-fold or higher) is observed for cysteine synthase (CysK, EC 2.5.1.47), which synthesizes cysteine from O-acetylserine and hydrogen sulfide (Figure 8, Table S3). It is possible that the upregulation of cysteine synthase was the key event that led to increased cysteine production due to the incorporation of inorganic sulfur from hydrogen sulfide.

Numerous studies have shown that adding methionine to fermentation medium stimulates biosynthesis of CPC in *A. chrysogenum* during the growth period [74,97,98]. In addition, methionine represses the utilization of organic sulfates. Experiments with L-methionine-*S*^35^ demonstrated that virtually all the sulfur in cephalosporins derived from methionine [97]. It was also shown that after random mutagenesis, *A. chrysogenum* strains with increased sulfate uptake and incorporation of inorganic sulfur into beta-lactams can be obtained [99]. The fermentation medium optimized for *A. chrysogenum* HY contains 14 g/L (NH_4_)_2_SO_4_, which corresponds to 3.4 g of sulfur, and can fully provide high-yielding production, based on the calculation that 0.77 g of sulfur are required to obtain 10 g of CPC. It is possible that the upregulation of cysteine synthase, as well as enzymes of the assimilatory sulfate reduction pathway, were the key events that led to increased cysteine production due to the incorporation of inorganic sulfur from hydrogen sulfide.

In the improved producer of *A. chrysogenum* HY, upregulation was found in the components of both cysteine biosynthesis pathways, from sulfate and from methionine. Perhaps such events are universal in nature, since a comparative proteomic analysis of strains sequentially obtained in the CSI program of *P. chrysogenum* (Wis 54-1255 (wild type) → Wis 54-1255 → ASP-78) showed that Wis 54-1255 had an upregulation of cysteine synthase, and the next strain in the improvement program, ASP-78, additionally upregulated the production of cystathionine gamma-lyase [40].

*A. chrysogenum* HY has significantly increased content, 1–4% of all proteins in the proteome, of thiamine-thiazole synthase (KFH43147.1, EC: 2.4.2.60), which is necessary for the synthesis of the highly active molecule thiamine pyrophosphate [80,81]. Thiamine pyrophosphate is vital coenzyme in carbohydrate metabolism, branched-chain amino acid, and other organic molecule metabolism, providing energy for cell metabolism [100,101]. This coenzyme is synthesized in the cytosol and is required in the cytosol for the activity of transketolase and in the mitochondria for the activity of pyruvate, oxoglutarate, and branched chain keto acid dehydrogenases [100,102]. Thiamine pyrophosphate has also been shown to have antioxidant activity and provide more effective protection against oxidative damage than thiamine [103,104,105,106]. In this regard, the increase in the content of thiamine-thiazole synthase in *A. chrysogenum* HY may be associated with the intensification of pathways providing energy for cell metabolism, where thiamine pyrophosphate serves as a cofactor for key enzymes. On the other hand, it can be involved in oxidative stress, where thiamine pyrophosphate is used as an antioxidant. Therefore, an increase in the thiamine-thiazole synthase protein content may indicate either that there has been an escape from riboswitch regulation, for example, due to alternative splicing, as has been shown for this gene in fungi [107,108], or that thiamine pyrophosphate is consumed to protect against oxidative stress and therefore does not inhibit the translation of its protein. In addition, eukaryotic thiamine thiazole synthase is a suicide enzyme. It undergoes only a single turnover, for which an iron-dependent sulfide transfer reaction from a conserved cysteine residue of the protein to a reaction intermediate has been demonstrated, leading to inactivation of the enzyme after catalysis [109]. Increased level of this enzyme in the *A. chrysogenum* HY proteomes may also be due to the detection of inactivated forms after one catalytic reaction by this suicidal single-turnover protein. Previously, it was shown that thiamine pyrophosphate biosynthesis is one of the key factors for the high-yield production of cephalosporin C (15 g/L and higher) in the improved *A. chrysogenum* strain 84-3-81-41 [41]. The data obtained in our work indicate universal changes occurring in independent CSI programs for high-yield production of cephalosporin C in *A. chrysogenum*.

Transketolase, which uses TPP as a cofactor [110], is one of the most important enzymes controlling cellular energy flow. This enzyme feeds excess sugar phosphates from the pentose phosphate pathway into glycolysis, which allows energy to be obtained in the form of ATP and NADPH [82]. An increase in the TK content of the HY strain by 5–10 times may indicate a reprogramming of energy flows for the needs of CPC biosynthesis (Figure 9). TK upregulation was also detected in the proteome of *P. chrysogenum* ASP-78, following the enhancement of Wis 54-1255 for PenG production [40]. The alterations that occurred in the SCI programs in such phylogenetically distant organisms as *A. chrysogenum* from the *Sordariomycetes* and *P. chrysogenum* from *Eurotiomycetes* may indicate the universality of the events necessary for high-yielding beta-lactam production, particularly at the level of the metabolic reprogramming, the withdrawal of excess sugar phosphates into the main carbohydrate metabolic pathways.

In the late stages of conidial differentiation, fungal cells produce a so-called conidiation-specific protein (Csp) [111]. In *A. chrysogenum* WT, the Csp protein (KFH48502.1) was detected after 120 h of fermentation, emPAI = 0.1143 (Table S2), which correlates with conidia formation at this stage of fermentation. In *A. chrysogenum* HY, the Csp was not detected at any stage of fermentation, which may be the reason for the lack of conidia formation in this strain. Since conidiation significantly increases the viability of fungal strains. In particular, it allows survival during low-temperature freezing, and the absence of the Csp protein (a marker for conidia formation) in the HY strain makes it possible to characterize these phenotypic differences at the molecular level.

One of the previously undescribed differences observed in comparative proteome analysis of the improved fungal strains was a significant upregulation of the Grg protein in the WT strain at the end of the fermentation process. The content of this protein reaches almost 40% of all cellular proteins and can be considered a marker of aging [112] (Table 1). In *A. chrysogenum* HY, this protein reaches is less than 1 percent after 96 h and is not detected after 120 h. Previously, it was shown that in *Neurospora crassa* the mRNA level for Grg increases sharply, up to 500-fold, within an hour under starvation conditions [113]. The transcription level depends on the characteristic upstream regulatory sites of CRE (GTGACGTCAC) and NRS (TTGCTAGCAA) [114]. In *Podospora anserina*, a mutation in the transcription factor Grisea leads to an increase in lifespan and leads to a decrease in the expression level of the *grg1* gene [112]. It is conceivable that alterations in the HY strain in addition to ensuring efficient production, enable the cells to maintain their youthfulness at the final stage of fermentation. It is possible that changes have occurred in the *A. chrysogenum* HY strain not only led to efficient production but also prevented cells from aging during the final stage of fermentation (Table 1, Figure 10).

The most represented protein in *A. chrysogenum* HY is peroxiredoxin, comprising 4–13% of the proteome; in the WT strain, this protein does not exceed 1% (Table 1). Among the other most represented proteins in *A. chrysogenum* HY, two CPC biosynthesis enzymes stand out: PcbC (0.6–1.9%) and CefEF (0.7–2.8%); in the WT strain, these proteins are present in tenths and hundredths of a percent. Also, the high-yielding producer contains an increased amount of the suicidal single-turn enzyme THI, up to 1–3.7%, which indicates active TPP synthesis.

**Figure 13:**
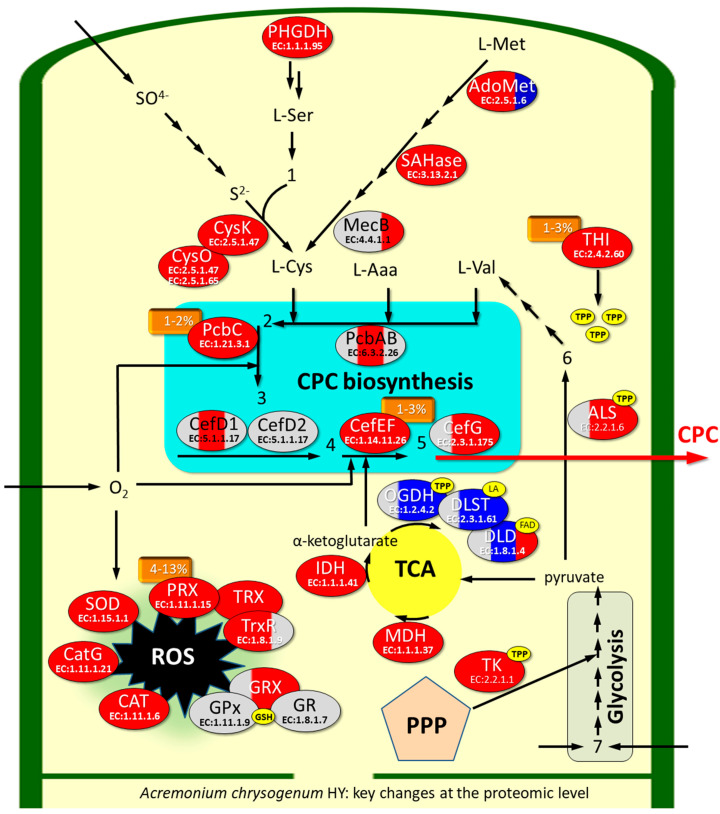
Key changes in the *A. chrysogenum* HY proteome for high-yield cephalosporin C (CPC) production revealed by comparative proteomic analysis with the wild-type parental strain *A. chrysogenum* WT. In *A. chrysogenum* HY, enzymes of the CPC biosynthesis pathway are upregulated, and metabolic pathways aimed at constructing precursor molecules are intensified. Specifically, enzymes involved in sulfur metabolism for cysteine production are upregulated, as is valine biosynthesis. The enzyme involved in α-ketoglutarate biosynthesis is also upregulated, while enzymes of the complex utilizing this metabolite in the tricarboxylic acid cycle (TCA) are downregulated. α-ketoglutarate is required for the expandase reaction of the CPC biosynthesis pathway. There is also a reprogramming of cellular energy flows in favor of the biosynthesis of CPC, covering the pentose phosphate pathway (PPP), glycolysis and TCA. Effective CPC production requires strong aeration, and in response to this, significant upregulation of oxidative stress enzymes occur. PHGDH—phosphoglycerate dehydrogenase (D-3-phosphoglycerate dehydrogenase, EC: 1.1.1.95), CySK—cysteine synthase 1 (EC: 2.5.1.47), CysO—cysteine synthase 2 (EC: 2.5.1.65), AdoMet—S-adenosylmethionine synthase (EC: 2.5.1.6), SAHase—Adenosylhomocysteinase (EC: 3.13.2.1), MecB—cystathionine gamma-lyase (EC: 4.4.1.1), THI—thiamine thiazole synthase (EC: 2.4.2.60), PcbAB—ACV (δ-[L-α-Aminoadipyl]-L-Cysteinyl-D-Valine) synthetase (EC: 6.3.2.26), PcbC—isopenicillin N-synthase (EC: 1.21.3.1), cefD1—isopenicillin N-CoA synthetase (EC: 5.1.1.17), cefD2—isopenicillin N-CoA epimerase (EC: 5.1.1.17), CefEF—deacetoxycephalosporin C synthetase (penicillin N expandase, EC: 1.14.20.1)/deacetoxycephalosporin C hydroxylase (EC:1.14.11.26), CefG—deacetylcephalosporin-C acetyltransferase (EC: 2.3.1.175), ALS—acetolactate synthase (EC: 2.2.1.6), SOD—superoxide dismutase (EC: 1.15.1.1), TRX—thioredoxin, TrxR—thioredoxin reductase (EC: 1.8.1.9), PRX—peroxiredoxin (EC: 1.11.1.15), GRX—glutaredoxin, GR—glutathione reductase (EC: 1.8.1.7), GPx—glutathione peroxidase (EC: 1.11.1.9), CAT—catalase (EC: 1.11.1.6), CatG—catalase-peroxidase (EC: 1.11.1.21), IDH—isocitrate dehydrogenase (EC 1.1.1.41), IDH—isocitrate dehydrogenase (EC 1.1.1.41), OGDH—oxoglutarate dehydrogenase (EC: 1.2.4.2), DLST—dihydrolipoyl succinyltransferase (EC: 2.3.1.61), DLD—dihydrolipoyl dehydrogenase (EC: 1.8.1.4), MDH—malate dehydrogenase (EC: 1.1.1.37), TK—transketolase (EC: 2.2.1.1). L-Aaa—L-α-aminoadipic acid, GSH—glutathione, TPP—thiamine phosphate, LA—lipoic acid, FAD—flavin adenine dinucleotide, 1–*O*-acetylserine, 2–LLD-ACV–δ-(L-α-aminoadipoyl)-L-cysteinyl-D-valine, 3–isopenicillin N, 4–penicillin N, 5–deacetylcephalosporin C, 6–α-acetolactic acid, 7–glucose. Upregulated enzymes in *A. chrysogenum* HY filled in red, downregulated filled in blue, which no distinctions filled in gray. Initial one third of the fill corresponds to 72 h of fermentation, the middle third, 96 h, and the last third, 120 h. Cofactors are filled in yellow. For the most represented proteins, the percentage of the proteome composition is shown in orange boxes.

The most important changes that occurred in *A. chrysogenum* HY at the proteome level are shown in Figure 13.

## 5. Conclusions

In this study, we performed a comparative proteomic analysis of the parental *A. chrysogenum* wild-type (WT) strain and obtained through CSI program *A. chrysogenum* HY, producing more than 10 g/L of antibiotic cephalosporin C during fermentation under submerged conditions. At different stages of fermentation, 30% or more differentially expressed proteins were observed. We aimed to identify the most important changes that led to high-yield CPC production. The key alteration appears to be the intensification of beta-lactam biosynthesis, manifested by upregulation of enzymes in this metabolic pathway. There was also a reprogramming of metabolic flows to supply the metabolism with the necessary “building blocks”. We identified upregulation of enzymes involved in the biosynthesis of valine and cysteine, which are required for the construction of the beta-lactam core structure. Upregulation of the enzymes involved in the biosynthesis of serine, a precursor in cysteine biosynthesis, and α-ketoglutarate, a substrate for the expandase reaction of the CPC biosynthesis pathway, was also observed. Moreover, the enzymes of the complex metabolizing α-ketoglutarate to succinyl-CoA in the tricarboxylic acid cycle were downregulated, potentially leading to α-ketoglutarate accumulation. Since sulfur was removed along with the cysteine molecule during the export of beta-lactams from the body, *A. chrysogenum* HY apparently underwent changes that allow it to effectively absorb sulfur in the form of sulfates and then incorporate it into the cysteine molecule. In support of this, we observed the upregulation of some enzymes of the assimilatory sulfate reduction pathway and cysteine synthases, which use the resulting hydrogen sulfide to synthesize cysteine.

Effective production of cephalosporin C requires strong aeration; in our study, we observed significant upregulation of oxidative stress enzymes, which may have been a response to increased oxygen concentrations, as described in previous comparative proteomics studies of improved beta-lactam producers. We also saw the alterations described for other high-yielding strains, leading to a reprogramming of energy flows in favor of targeted metabolism. In particular, we noted upregulation of transketalase, which removes sugars from the pentose phosphate pathway for the energy-producing processes of glycolysis, and then the tricarboxylic acid cycle. We also observed a significant upregulation of thiamine-thiazole synthase, which synthesizes the transketalases cofactor thiamine pyrophosphate, TPP. The obtained data confirm and expand the knowledge about changes at the proteome level occurring in programs for improving fungal strains for high-yield production of secondary metabolites and beta-lactams, in particular. This is important both for understanding the molecular basis of the processes that occurred and for the targeted engineering of high-yield producers of pharmaceutically significant secondary metabolites.

## Figures and Tables

**Table 1:** Content of selected proteins (%) in the proteomes of *A. chrysogenum* WT and HY strains after submerged fermentation on complex CP medium for 72, 96, and 120 h.

Protein	GenBank Accession №:	Protein Content of the Proteome, %					
		WT 72	WT 96	WT 120	HY 72	HY 96	HY 120
Isopenicillin N-synthase (PcbC)	KFH48817.1	N/D *	0.11	N/D	1.87	1.18	0.57
PenN expandase/DAOC hydroxylase (CefEF)	KFH44919.1	N/D	0.06	N/D	2.77	0.97	0.72
Thiamine thiazole synthase (THI)	KFH43147.1	0.79	0.21	N/D	1.75	1.05	3.66
Peroxiredoxin (PRX)	KFH47331.1	0.72	0.63	1.1	6.51	3.93	13.25
Glucose-repressible gene (Grp)	KFH46345.1	1.25	8.29	37.57	N/D	0.25	N/D

*—no data.

## Data Availability

The proteomics data have been uploaded to Mendeley Data; accession DOI: 10.17632/p6977sbwkm.1, link: https://data.mendeley.com/datasets/p6977sbwkm/1 (accessed on 15 October 2025). The original contributions presented in this study are included in the article and Supplementary Material. Further inquiries can be directed to the corresponding author.

## Supplementary Materials

The following supporting information can be downloaded at: https://www.mdpi.com/article/10.3390/jof11110822/s1. Table S1: Data after proteomic analysis by LC-MS/MS of samples isolated from *A. chrysogenum* HY and WT strains after 72, 96 and 120 h of fermentation; Table S2: emPAI of individual proteins within proteomes, normalized to GAPDH; Table S3: Hierarchical cluster analysis of DEPs in *A. chrysogenum* WT and HY strains; Table S4: Enrichment analysis of overrepresented GO terms in DEPs in *A. chrysogenum* strains (HY vs. WT); Table S5: Hierarchical cluster analysis of DEPs in *A. chrysogenum* WT during fermentation and in *A. chrysogenum* HY during fermentation; Table S6: Enrichment analysis of overrepresented GO terms in DEPs in *A. chrysogenum* WT (96/72 and 120/72 h) and in *A. chrysogenum* HY (96/72 and 120/72 h).

## Abbreviations

The following abbreviations are used in this manuscript:

1,3-DAP	1,3-diaminopropane
Acetyl-CoA	Acetyl-coenzyme A
AdoMet	S-adenosylmethionine synthase
ALS	Acetolactate synthase
ATMT	*Agrobacterium tumefaciens*-mediated transformation
BGC	Biosynthetic gene cluster
CAA	Agarized Czapek-L-Asn (medium)
CAT	Catalase
CatG	Catalase-peroxidase
CDA	Agarized Czapek-Dox (medium)
CefD1	Isopenicillin N-CoA synthetase
CefD2	Isopenicillin N-CoA epimerase
CefEF	Deacetoxycephalosporin C synthetase (penicillin N expandase)/deacetoxycephalosporin C hydroxylase
CefG	Deacetylcephalosporin-C acetyltransferase
CP	Complex (medium)
CPA	Agarized complex (medium)
CPC	Cephalosporin C
COX	Cytochrome c oxidase
CSI	Classical strain improvement
CysK	Cysteine synthase 1
CysO	Cysteine synthase 2
DAC	Deacetylcephalosporin C
DAOC	Deacetoxycephalosporin C
DEPs	Differentially expressed proteins
DLD	Dihydrolipoyl dehydrogenase
DLST	Dihydrolipoyl succinyltransferase
DTT	Dithiothreitol
GPx	Glutathione peroxidase
GR	Glutathione reductase
Grg	Glucose-repressible gene
GRX	Glutaredoxin
HY	High-yielding
IDH	Isocitrate dehydrogenase
IPN	Isopenicillin N
LLD-ACV	δ-(L-α-aminoadipoyl)-L-cysteinyl-D-valine
MDH	Malate dehydrogenase
MecB	Cystathionine gamma-lyase
MMSDH	Methylmalonate-semialdehyde dehydrogenase
ODC	Ornithine decarboxylase
OGDH	Oxoglutarate dehydrogenase
PAs	Polyamines
PcbAB	ACV (δ-[L-α-Aminoadipyl]-L- Cysteinyl-D-Valine) synthetase
PcbC	Isopenicillin N-synthase
PenG	Penicillin G
PenN	Penicillin N
PHGDH	Phosphoglycerate dehydrogenase
PKS	Polyketide synthases
PRX	Peroxiredoxin
SAHase	Adenosylhomocysteinase
SAM	S-adenosylmethionine
SAMase	SAM-dependent methylase
SN	Synthetic (medium)
SOD	Superoxide dismutase
SPD	Spermidine
ROS	Reactive oxygen species
TCA	Trichloroacetic acid
THI	Thiamine thiazole synthase
TK	Transketolase
TPP	Thiamine pyrophosphate
TRX	Thioredoxin
TrxR	Thioredoxin reductase
WT	Wild type
